# Replacement of Dietary Saturated Fat by PUFA-Rich Pumpkin Seed Oil Attenuates Non-Alcoholic Fatty Liver Disease and Atherosclerosis Development, with Additional Health Effects of Virgin over Refined Oil

**DOI:** 10.1371/journal.pone.0139196

**Published:** 2015-09-25

**Authors:** Martine C. Morrison, Petra Mulder, P. Mark Stavro, Manuel Suárez, Anna Arola-Arnal, Wim van Duyvenvoorde, Teake Kooistra, Peter Y. Wielinga, Robert Kleemann

**Affiliations:** 1 Department of Metabolic Health Research, Netherlands Organisation for Applied Scientific Research (TNO), Leiden, the Netherlands; 2 Department of Pathology and Medical Biology, University of Groningen, University Medical Center Groningen, Groningen, the Netherlands; 3 Bunge Ltd., White Plains, New York, United States of America; 4 Department of Biochemistry and Biotechnology, Rovira iVirgili University, Tarragona, Spain; 5 Centre Tecnològic de Nutrició i Salut (CTNS), TECNIO, CEICS, Reus, Spain; 6 Department of Human and Animal Physiology, Wageningen University, Wageningen, the Netherlands; Wake Forest School of Medicine, UNITED STATES

## Abstract

**Background and Aims:**

As dietary saturated fatty acids are associated with metabolic and cardiovascular disease, a potentially interesting strategy to reduce disease risk is modification of the quality of fat consumed. Vegetable oils represent an attractive target for intervention, as they largely determine the intake of dietary fats. Furthermore, besides potential health effects conferred by the type of fatty acids in a vegetable oil, other minor components (e.g. phytochemicals) may also have health benefits. Here, we investigated the potential long-term health effects of isocaloric substitution of dietary fat (i.e. partial replacement of saturated by unsaturated fats), as well as putative additional effects of phytochemicals present in unrefined (virgin) oil on development of non-alcoholic fatty liver disease (NAFLD) and associated atherosclerosis. For this, we used pumpkin seed oil, because it is high in unsaturated fatty acids and a rich source of phytochemicals.

**Methods:**

ApoE*3Leiden mice were fed a Western-type diet (CON) containing cocoa butter (15% w/w) and cholesterol (1% w/w) for 20 weeks to induce risk factors and disease endpoints. In separate groups, cocoa butter was replaced by refined (REF) or virgin (VIR) pumpkin seed oil (comparable in fatty acid composition, but different in phytochemical content).

**Results:**

Both oils improved dyslipidaemia, with decreased (V)LDL-cholesterol and triglyceride levels in comparison with CON, and additional cholesterol-lowering effects of VIR over REF. While REF did not affect plasma inflammatory markers, VIR reduced circulating serum amyloid A and soluble vascular adhesion molecule-1. NAFLD and atherosclerosis development was modestly reduced in REF, and VIR strongly decreased liver steatosis and inflammation as well as atherosclerotic lesion area and severity.

**Conclusions:**

Overall, we show that an isocaloric switch from a diet rich in saturated fat to a diet rich in unsaturated fat can attenuate NAFLD and atherosclerosis development. Phytochemical-rich virgin pumpkin seed oil exerts additional anti-inflammatory effects resulting in more pronounced health effects.

## Introduction

Cardiometabolic diseases such as non-alcoholic fatty liver disease (NAFLD) and cardiovascular disease (CVD) constitute a major health burden in modern societies. Accumulating evidence suggests that NAFLD, besides increasing liver morbidity and mortality, is associated with development of atherosclerosis, the major underlying pathology of CVD [1]. As dyslipidaemia and chronic inflammation are recognised to drive the development of NAFLD as well as atherosclerosis [2–4], dietary regimens that influence one or both of these risk factors may be of great preventive and possibly even therapeutic benefit. Support for this concept comes from epidemiological and experimental studies that show that the type of dietary fat consumed plays an important role in the development of both NAFLD and associated CVD (reviewed in [5, 6]). Therefore, a potentially interesting strategy to reduce cardiometabolic risk is a modification of the quality of fat in diets. This is further supported by results from a recent systematic review indicating that partial replacement of saturated fat by unsaturated fat may reduce CVD risk [7].

The daily intake of dietary fats is largely determined by vegetable oils, which makes them an attractive target for intervention. The more so, since besides potential health effects conferred by the type of fatty acids in a vegetable oil, other minor components of an oil (e.g. phytochemicals) may also significantly contribute to cardiometabolic health. Typically, vegetable oils are consumed in their fully refined form that consists almost exclusively of triglycerides. Virgin oils on the other hand, the completely unrefined first press form of an oil, are rich in a collection of phytochemicals (e.g. vitamins E and K, phytosterols and polyphenols) that may influence the critical risk factors dyslipidaemia as well as inflammation [8, 9].

Herein we investigated the potential long-term health effects of substitution of dietary saturated fat by unsaturated fat from refined oil, as well as putative additional effects of the unrefined counterpart rich in phytochemicals (virgin oil). For this, we used pumpkin seed oil, because it is high in unsaturated fatty acids (about 80%) and known to contain large amounts of phytochemicals [10, 11]. In short-term studies, pumpkin seed oil has been shown to reduce surrogate markers of liver health [12] and improve dyslipidaemia [13–15]. However, potential anti-inflammatory properties have not been examined and its effects on cardiometabolic disease endpoints are unknown.

The ApoE*3Leiden (E3L) mouse is a well-established diet-inducible model for NAFLD [16] and atherosclerosis [17]. The model develops human-like dyslipidaemia, inflammation and disease endpoints in response to a well-defined Western-type diet, containing cocoa butter (±60% saturated fat) as the major fat source [17, 18]. This diet also contains cholesterol (1% w/w), which is required for induction of dyslipidaemia, inflammation and disease endpoints [16, 18, 19]. Groups of E3L mice were fed the Western-type control diet (CON) or pumpkin seed oil-substituted diets, REF (refined oil) and VIR (virgin oil) for 20 weeks, all of which contained 1% cholesterol. The refined and virgin pumpkin seed oils were comparable in fatty acid profile but differed in phytochemical content. This allowed us to define the health effects of refined pumpkin seed oil, as well as the additional effects of the phytochemicals present in its unrefined counterpart. Plasma lipids and markers of inflammation were monitored over time and NAFLD and atherosclerosis endpoints were scored according to established human grading systems ([20–22]). Results from this study indicate that an isocaloric switch from a diet rich in saturated fat to a diet rich in unsaturated fat has beneficial effects on risk factors, and that phytochemical-rich virgin oil has additional anti-inflammatory properties and more strongly reduces disease endpoints.

## Materials and Methods

All animal experiments were approved by an independent Ethical Committee on Animal Care and Experimentation (DEC-Zeist, the Netherlands) and were in compliance with European Community specifications regarding the use of laboratory animals. Female ApoE*3Leiden transgenic (E3L) mice were obtained from the breeding facility of TNO Metabolic Health Research, Leiden, the Netherlands, and were characterised for expression of human APOE by ELISA. 12-week old E3L mice were matched into 3 groups based on plasma cholesterol and triglycerides. All animals were group-housed (3–4 mice per cage) in the SPF animal facility of TNO Metabolic Health Research, in a temperature-controlled room on a 12 hour light/dark cycle and had free access to food and water. Diets were based on a standardised atherogenic Western-type diet (WTD) that contains 15% cocoa butter, 1% corn oil, 40.5% sucrose, 20% acid casein, 10% corn starch and 6.2% cellulose (all w/w; diet-T; AB-Diets, Woerden, the Netherlands), supplemented with 1% (w/w) cholesterol (Sigma-Aldrich, Zwijndrecht, the Netherlands). Control mice (CON, n = 18) were fed this standard WTD, while the treatment groups received the WTD with 9% (w/w of total diet) of the cocoa butter replaced by either 9% refined pumpkin seed oil (REF, n = 15; Bunge Ltd., White Plains, USA) or 9% virgin pumpkin seed oil (VIR, n = 15; Bunge Ltd). As the cholesterol in this diet is required to induce inflammation and dyslipidaemia [16, 18, 19], the cholesterol concentration was the same (1%) in all three groups.

Detailed methods of the analysis of the composition of the cocoa butter and pumpkin seed oils are described in S1 Text. Briefly, the fatty acid composition was determined by gas chromatography, the total phenolic content was determined spectrophotometrically by the Folin-Ciolcalteau method, and individual phenolic content of the pumpkin seed oils was determined by LC-QTOF-MS.

Food intake was measured per cage (3–4 mice per cage) every 4 weeks, expressed as the average food intake per mouse per day. The energy content of the diets was determined by bomb calorimetry. Blood samples were collected from the tail vein after a 4h fasting period for EDTA plasma isolation at week 0, 3, 6, 12 and 20 of the study. Total plasma cholesterol and triglyceride levels were measured in these fasted plasma samples by commercially available enzymatic assays (cholesterol CHOD-PAP 11491458 and triglycerides GPO-PAP 11488872, Roche, Woerden, The Netherlands). For lipoprotein profile analysis, pooled plasma samples were fractionated using an ÅKTA fast protein liquid chromatography system (Pharmacia, Roosendaal, the Netherlands) and analysed as reported [23]. Plasma levels of soluble vascular adhesion molecule 1 (sVCAM-1; R&D Systems, Abingdon, UK) and serum amyloid A (SAA; Life Technologies, Bleiswijk, the Netherlands) were determined by ELISA. ALAT and ASAT levels were measured in serum (unfasted sample from terminal blood, specified below) using a spectrophotometric activity assay (Reflotron Plus system, Roche). After 20 weeks of dietary treatment, mice were sacrificed by CO_2_ asphyxiation and blood was collected via cardiac puncture for serum collection (unfasted). Hearts and livers were collected, and fixed in formalin and embedded in paraffin for atherosclerosis analysis (heart) and NAFLD analysis (liver).

### Histological analysis of NAFLD and atherosclerosis development

For NAFLD analysis, 3 μm liver sections (medial lobe) were stained with haematoxylin and eosin and analysed blindly using an adapted scoring method for human NAFLD [20, 24]. Briefly, steatosis was expressed as the percentage of the total liver cross section affected by microvesicular steatosis or macrovesicular steatosis. Hepatic inflammation was analysed by counting the number of inflammatory foci per section at a 100× magnification.

Atherosclerosis was analysed blindly in 4 serial cross sections (5 μm, at 50 μm intervals) of the valve area of the aortic root. Cross sections were stained with haematoxylin-phloxine-saffron (HPS) for morphometric analysis of lesion number and area (using cell^D software, version 2.7; Olympus Soft Imaging Solutions, Hamburg, Germany) and analysis of lesion severity. Lesion severity was scored according to the classification of the American Heart Association (AHA) [21, 22]. This scoring system was used to distinguish five lesion types: Type I (early fatty streak): up to ten foam cells in the intima, no other changes; Type II (regular fatty streak): ten or more foam cells in the intima, no other changes; Type III (mild plaque): foam cells in the intima with presence of a fibrotic cap; Type IV (moderate plaque): progressive lesion, infiltration into media, elastic fibres intact; Type V (severe plaque): structure of media severely disrupted with fragmented elastic fibres, cholesterol crystals, calcium deposits and necrosis may be present. The lesional macrophage content was assessed by immunohistochemical staining of MAC-3 (CD107b) positive cells (purified rat anti-mouse CD107b antibody, BD Biosciences, Breda, the Netherlands) in cross-sections adjacent to those used for the atherosclerosis analysis. The MAC-3 positive area for each individual plaque was measured using an automated macro in the image processing software ImageJ (version 1.48, NIH, Bethesda, MD, USA; [25]) and expressed as the percentage of total plaque area that was positively stained for MAC-3. The number of lesions was counted in 4 cross sections and expressed as the average per cross-section. Furthermore, the number of lesion-free (undiseased) segments was counted and expressed as a percentage of the total number of segments (N.B. each aortic cross-section is divided into 3 segments that are demarcated by the aortic valves, making a total of 12 segments analysed per mouse).

### Hepatic gene expression analyses

Total RNA was extracted from liver tissue using RNA Bee Total RNA Isolation Kit (Bio-Connect, Huissen, the Netherlands). Spectrophotometric analysis of RNA concentration was performed using Nanodrop 1000 (Isogen Life Science, De Meern, the Netherlands) and quality of RNA was assessed using a 2100 Bioanalyzer (Agilent Technologies, Amstelveen, the Netherlands). cDNA was synthesised using a High Capacity RNA-to-cDNA™ Kit (Life Technologies, Bleiswijk, the Netherlands). Hepatic gene expression analyses were performed by RT-PCR on a 7500 Fast Real-Time PCR System (Applied Biosystems by Life Technologies) using TaqMan® Gene Expression Assays (Life Technologies). Transcripts were quantified using TaqMan® Gene Expression Assays (Life Technologies) and the following primer/probe-sets for *Srebf1* (Mm00550338_m1), *Fasn* (Mm00662319_m1), *Dgat1* (Mm00515643_m1), *Ppara* (Mm00440939_m1), *Cpt1a* (Mm01231183_m1), *Acox1* (Mm00443579_m1), *Ccl2* (Mm00441242_m1), *Tnf* (Mm00443258_m1), *Il1b* (Mm00434228_m1) and the endogenous controls *Hprt* (Mm00446968_m1) and *Ppif* (Mm01273726_m1). Changes in gene expression were calculated using the comparative Ct (ΔΔCt) method and expressed as fold-change relative to CON.

### Hepatic lipid analysis

Lipids were extracted from liver homogenates using the Bligh and Dyer method [26] and separated by high performance thin layer chromatography (HPTLC) on silica gel plates as described previously [27]. Lipid spots were stained with color reagent (5g MnCl_2_·4H_2_O, 32ml 95–97% H_2_SO_4_ added to 960ml CH_3_OH:H_2_O 1:1 v/v) and triglycerides, cholesteryl esters and free cholesterol were quantified using TINA version 2.09 software (Raytest, Straubenhardt, Germany).

### Statistical analyses

All data are presented as mean±SEM. Statistical analyses were performed using SPSS software (version 22, IBM, Armonk, USA). For normally distributed variables, significance of differences between groups was tested by one-way ANOVA, followed by Fisher's Least Significant Difference (LSD) Post-Hoc Test. In case of heterogeneity between groups, variables were analysed by ANOVA using Brown-Forsythe for differences between groups followed by Dunnett’s T3 Post-Hoc Test. Non-normally distributed variables were tested by non-parametric Kruskal-Wallis test followed by Mann-Whitney U tests. To test the hypothesis that both pumpkin seed oils may have beneficial effects relative to control and that the virgin oil may have additional beneficial effects over its refined counterpart, a one-sided p-value≤0.05 was considered statistically significant.

## Results

The refined and virgin pumpkin seed oils used in this study were comparable in fatty acid composition (Table 1). Both oils contained 81% unsaturated fatty acids, most of which consisted of linoleic acid (C18:2n-6, 64%) and oleic acid (C18:1n-9, 17%). The virgin oil contained more phytochemicals than its refined counterpart (Table 2). Virgin pumpkin seed oil was rich in benzoic acid, vanillic acid, ferulic acid, rutin and *p*-coumaric acid, many of which were below the detection limit in the refined oil. Overall, the total phenolic content was 7.7-fold higher in the virgin oil than in the refined oil.

**Table 1:** Fatty acid composition of cocoa butter and refined and virgin pumpkin seed oil.

		Cocoa butter	Refined pumpkin seed oil	Virgin pumpkin seed oil
**Poly-unsaturated fatty acids** (% of total)		**2.8**	**64.4**	**64.0**
C18:2	Linoleic acid (n-6)	2.7	64.1	63.9
C18:3	alpha-Linolenic acid (n-3)	0.1	0.3	0.1
**Mono-unsaturated fatty acids** (% of total)		**33.0**	**17.0**	**17.8**
C18:1	Oleic acid	32.8	16.6	17.1
C16:1	Palmitoleic acid	0.2	0.3	0.2
C20:1	Eicosanoic acid	n.d.	0.1	0.4
**Saturated fatty acids** (% of total)		**63.7**	**18.0**	**18.1**
C16:0	Palmitic acid	26.7	12.8	12.8
C18:0	Stearic acid	35.7	4.5	4.5
C20:0	Arachidic acid (Eicosanoic acid)	1.0	0.3	0.3
C22:0	Behenic acid (Docosanoic acid)	0.2	0.2	0.2
C14:0	Myristic acid (Tetradecanoic acid)	0.1	0.1	0.1
C24:0	Lignoceric acid (Tetracosanoic acid)	n.d.	0.1	0.1
**Trans fatty acids** (% of total)		**n.d**.	**0.7**	**0.2**
C18:2T	Trans linoleic acid	n.d.	0.7	0.2

n.d. = not detected.

**Table 2:** Phytochemical content of cocoa butter and refined and virgin pumpkin seed oil

	Cocoa butter	Refined pumpkin seed oil	Virgin pumpkin seed oil
Tocopherols (ppm)	246	386	577
Tocotrienols (ppm)	7	123	121
Vitamin K (μg/100g)	3.5	52.3	68.0
Total phenolic content (mg gallic acid/kg oil)	8.3	3.6	27.7
Benzoic acid (μM)	n.d.	0.1	19.3
*p*-coumaric acid (nM)	n.d.	n.d.	200
Vanillic acid (nM)	791	n.d.	300
Ferulic acid (nM)	n.d.	n.d.	300
Rutin (nM)	n.d.	n.d.	4
Isomer of 3-hydroxybenzoic acid (nM)	n.d.	50	8000
Isomer of protocatechuic acid (nM)	n.d.	n.d.	700
Isomer of caffeic acid (nM)	n.d.	n.d.	60
Isomer of ferulic acid (nM)	456	30	100
Isomer of naringenin (nM)	162	n.d.	2100
Isomer of 4-hydroxyphenylpropionic (nM)	522	10	700

n.d. = not detected, Tocopherols = sum of α, β, γ and δ (δ was n.d.). Tocotrienols = sum of α, γ and δ.

To investigate potential health effects of these oils on NAFLD and atherosclerosis, E3L mice were fed a standardised Western type control diet (CON) or the same diet substituted with 9% (w/w) refined pumpkin seed oil (REF) or 9% (w/w) virgin pumpkin seed oil (VIR) for 20 weeks. All diets contained 1% (w/w) cholesterol and were comparable in energy content as quantified by bomb calorimetry (CON: 20.2 kJ/g, REF: 20.0 kJ/g and VIR: 20.4 kJ/g) and food intake was comparable between groups (S1 Fig). The treatments were well tolerated and body weight increased slightly over time (percentage body weight gain relative to t = 6: CON: 12.3±1.3%, REF: 8.9±1.8%, VIR: 11.1±1.6%, n.s.) in all groups (S1 Fig).

### Both pumpkin seed oils improve dyslipidaemia, with additional beneficial effects of virgin oil over refined oil

**Fig 1:**
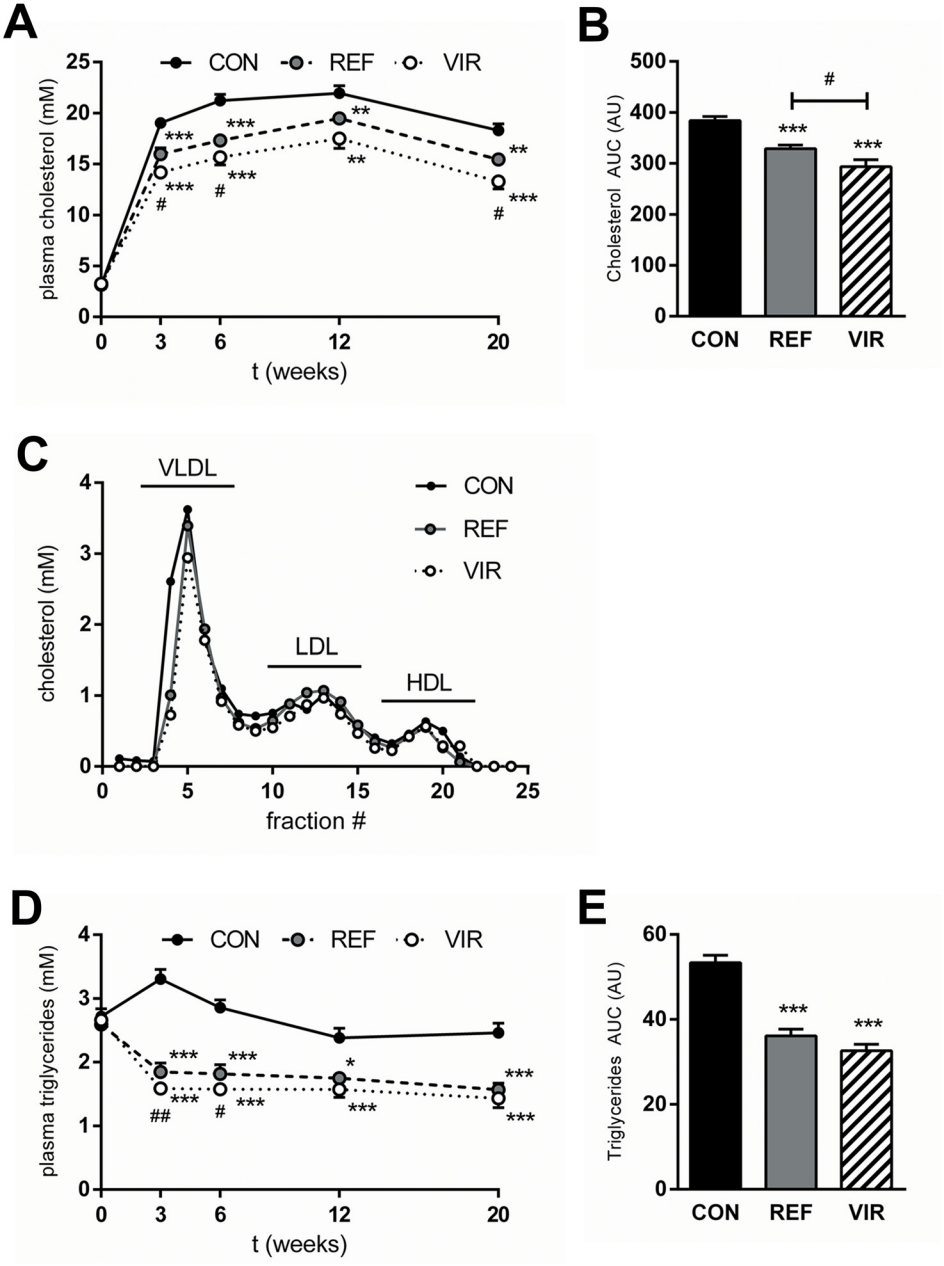
Refined and virgin pumpkin seed oils have beneficial effects on plasma lipids in cholesterol-fed ApoE*3Leiden mice. Mice were fed a Western type diet (CON) containing cocoa butter (15% w/w of diet) for 20 weeks. The cocoa butter was in part replaced by refined pumpkin seed oil (REF) or virgin pumpkin seed oil (VIR) (each 9% w/w of diet). A: Plasma cholesterol levels over the course of the study, showing lower levels in REF and VIR-fed animals. B: Area under the curve analysis (AUC, expressed in arbitrary units; AU) of plasma cholesterol levels (t = 0 until t = 20 weeks) shows additional cholesterol-lowering effect of VIR compared with REF. C: Lipoprotein profile for cholesterol distribution in VLDL, LDL and HDL-sized particles shows cholesterol-lowering effect mainly confined to VLDL-sized particles. D: Plasma triglycerides over the course of the study were lowered by both REF and VIR. E: Area under the curve analysis of plasma triglyceride levels (t = 0 until t = 20 weeks) shows a reduction by VIR and REF. Data are mean±SEM. * p≤0.05, ** p≤0.01, *** p≤0.001 compared with CON. # p≤0.05, ## p≤0.01 for VIR compared with REF.

Plasma cholesterol levels rose rapidly in CON animals within the first 3 weeks and remained relatively stable until the end of the study (Fig 1a) with an average of 19.20±0.39 mM. Both REF and VIR animals had significantly lower fasting plasma cholesterol levels compared with CON at all time points (Fig 1a). Area under the curve (AUC) analysis of the plasma cholesterol levels throughout the study period showed a significantly lower AUC for cholesterol in VIR (293.6±13.6 AU),than in REF (328.8±7.0 AU, p≤0.05, Fig 1b), indicating additional cholesterol-lowering properties of VIR. These cholesterol-lowering effects were mainly confined to the VLDL-sized particles (Fig 1c). In CON animals, fasting plasma triglycerides remained at a stable and elevated level during the study (average 2.67±0.09 mM, (Fig 1d). Both pumpkin seed oils decreased fasting plasma triglyceride levels within the first 3 weeks of the study and levels remained stable at this low level thereafter (average REF 1.79±0.08 mM, average VIR 1.63±0.07 mM, Fig 1d). Overall, VIR treatment did not have additional beneficial effects on plasma triglyceride levels relative to REF as is also shown by results from the AUC analysis for plasma triglyceride levels (Fig 1e). Together, these results indicate that the observed lipid-lowering effects are predominantly attributable to the replacement of saturated by unsaturated dietary fat.

### Virgin pumpkin seed oil reduces circulating markers of liver and vascular inflammation

**Fig 2:**
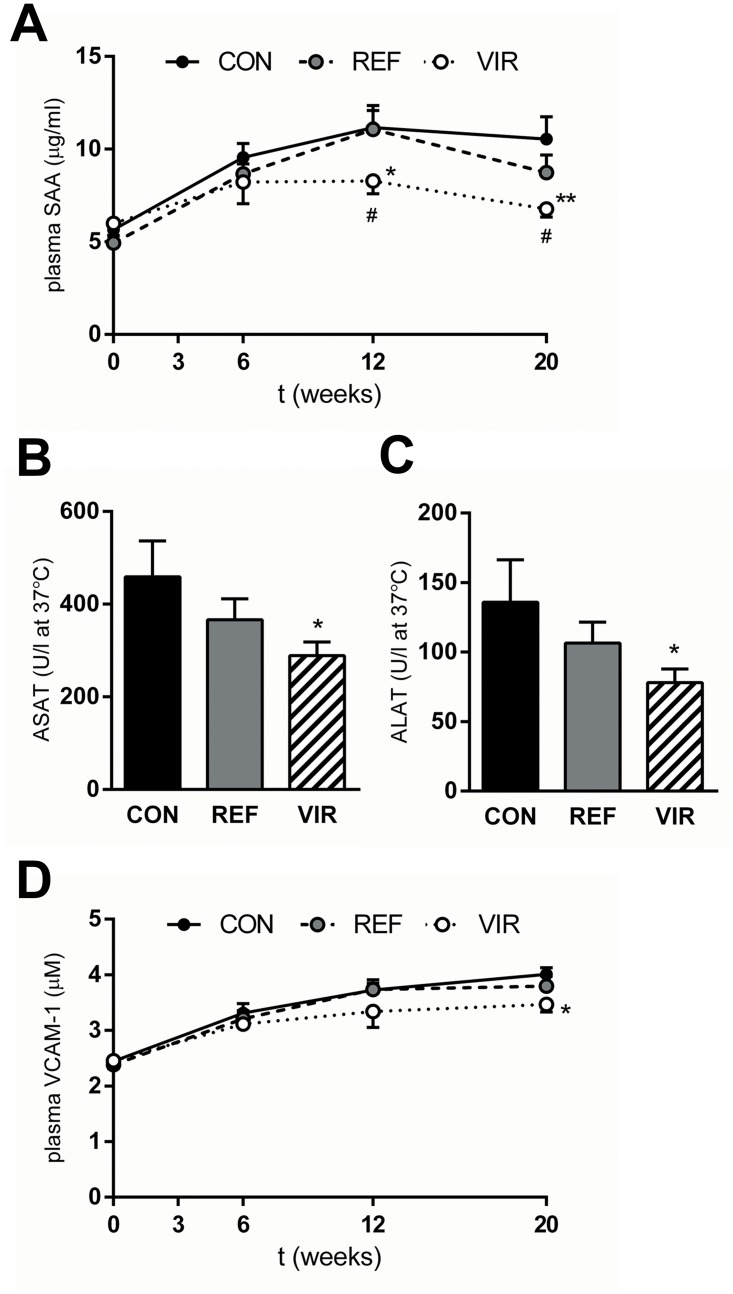
Virgin pumpkin seed oil reduces circulating markers of inflammation in cholesterol-fed ApoE*3Leiden mice. Mice were fed a Western type control diet (CON) or CON diet containing 9% refined pumpkin seed oil (REF) or 9% virgin pumpkin seed oil (VIR) for 20 weeks. A: Plasma SAA levels were reduced by VIR. Liver damage marker B: ASAT and C: ALAT were reduced by VIR but not by REF. D: Plasma sVCAM-1 levels in VIR animals were lower throughout the duration of the study. Data are mean±SEM. * p≤0.05, ** p≤0.01 compared with CON. # p≤0.05 for VIR compared with REF.

CON diet induced plasma levels of SAA, a marker of liver inflammation, from 5.65±0.34 μg/ml at t = 0 to 10.55±1.21 μg/ml at the end of the study (Fig 2a). SAA levels in REF animals were comparable to CON, while VIR attenuated SAA induction and plasma levels were significantly lower than CON at t = 12 and t = 20 weeks (6.77±0.44 μg/ml at t = 20, -36%, p≤0.01, Fig 2a). In line with this effect on SAA, serum levels of the hepatocellular damage markers ASAT and ALAT were not affected by REF, and VIR significantly reduced both ASAT (p≤0.05) and ALAT levels (p≤0.05) (Fig 2b and 2c). Besides inducing liver inflammation, CON diet also gradually induced plasma levels of vascular inflammation marker sVCAM-1 from 2.45±0.09 μg/ml at t = 0 to 4.01±0.12 μg/ml at t = 20 weeks (Fig 2d). Levels of sVCAM-1 were not affected by REF, but VIR animals showed lower sVCAM-1 throughout the study period and this effect reached significance at t = 20 weeks (3.47±0.14 μg/ml, -14%, p≤0.05, Fig 2d). These data show that the phytochemicals in virgin pumpkin seed oil are responsible for the observed anti-inflammatory effects on circulating liver and vascular inflammation markers.

### Virgin pumpkin seed oil attenuates development of NAFLD

**Fig 3:**
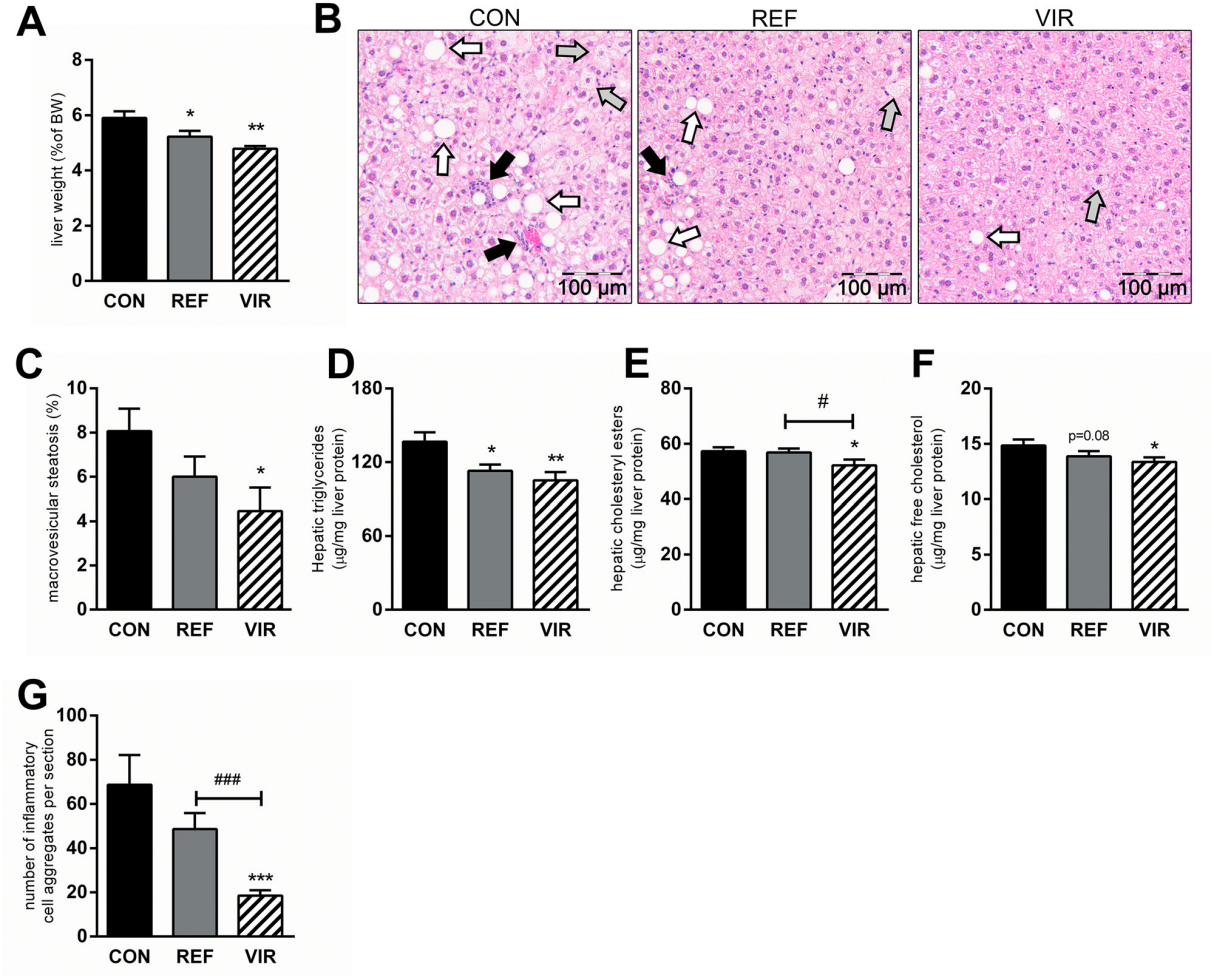
Virgin pumpkin seed oil attenuates development of NAFLD in cholesterol-fed ApoE*3Leiden mice. Mice were fed a Western type control diet (CON) or CON diet containing 9% refined pumpkin seed oil (REF) or 9% virgin pumpkin seed oil (VIR) for 20 weeks. A: Liver weight (expressed as percentage of terminal body weight) was reduced by REF and VIR. B: representative photomicrographs of HE-stained liver sections show presence of micro- (grey arrows) and macro- (white arrows)vesicular steatosis and inflammatory cell clusters (black arrows) in CON-fed animals, which was less pronounced in REF and more strongly reduced in VIR. C: Histological quantitative scoring of macrovesicular steatosis showed significant reduction in VIR. D: Hepatic triglyceride levels (biochemically determined) were reduced in both REF and VIR while only VIR significantly reduced E: hepatic cholesteryl ester content and F: free (unesterified) cholesterol levels. G: Histological quantification of number of inflammatory cell aggregates revealed a significant attenuation of hepatic inflammation by VIR. Data are mean±SEM. * p≤0.05, ** p≤0.01, *** p≤0.001 compared with CON. # p≤0.05, ### p≤0.001 for VIR compared with REF.

Refined pumpkin seed oil reduced liver weight by 12% (CON: 5.9±0.2% of terminal body weight, REF: 5.2±0.2%, p≤0.05, Fig 3a) and this effect was even stronger in VIR (-19%), with liver weights reduced to 4.8±0.1% of terminal body weight (p≤0.01, Fig 3a). Histological examination of the livers from CON animals revealed that NAFLD developed in these mice up to the stage of non-alcoholic steatohepatitis (NASH). CON mice displayed distinctive morphological hallmarks of NASH (pronounced steatosis and lobular infiltration of inflammatory cells) and the observed pathology was less severe in REF and VIR animals (representative photomicrographs shown in Fig 3b). Quantitative scoring of NAFLD revealed that macrovesicular steatosis tended to be lower in REF (-26%, p = 0.08) and was significantly reduced with VIR (-45%, p≤0.01) (Fig 3c). Microvesicular steatosis was less pronounced in both REF and VIR (-41% and -65%, respectively), but this effect did not reach statistical significance (S2 Fig). Biochemical analysis of hepatic lipid levels confirmed the histologically observed antisteatotic effects of the pumpkin seed oils, showing reduced hepatic triglyceride content in both REF and VIR (-17%, p≤0.01 and -23%, p≤0.001 respectively, Fig 3d). Hepatic cholesterol levels, in both esterified (Fig 3e) and unesterified (Fig 3f) form, were affected only in VIR, with slightly but statistically significantly reduced levels of these lipid species in this group. Consistent with the observed effects on plasma inflammation markers, infiltration of inflammatory cells was moderately lowered by REF (-29%, n.s.), while VIR strongly and significantly reduced lobular inflammation (-73%, p≤0.001 vs CON, p≤0.001 vs REF; Fig 3g).

### Atherosclerosis development is reduced with virgin pumpkin seed oil

**Fig 4:**
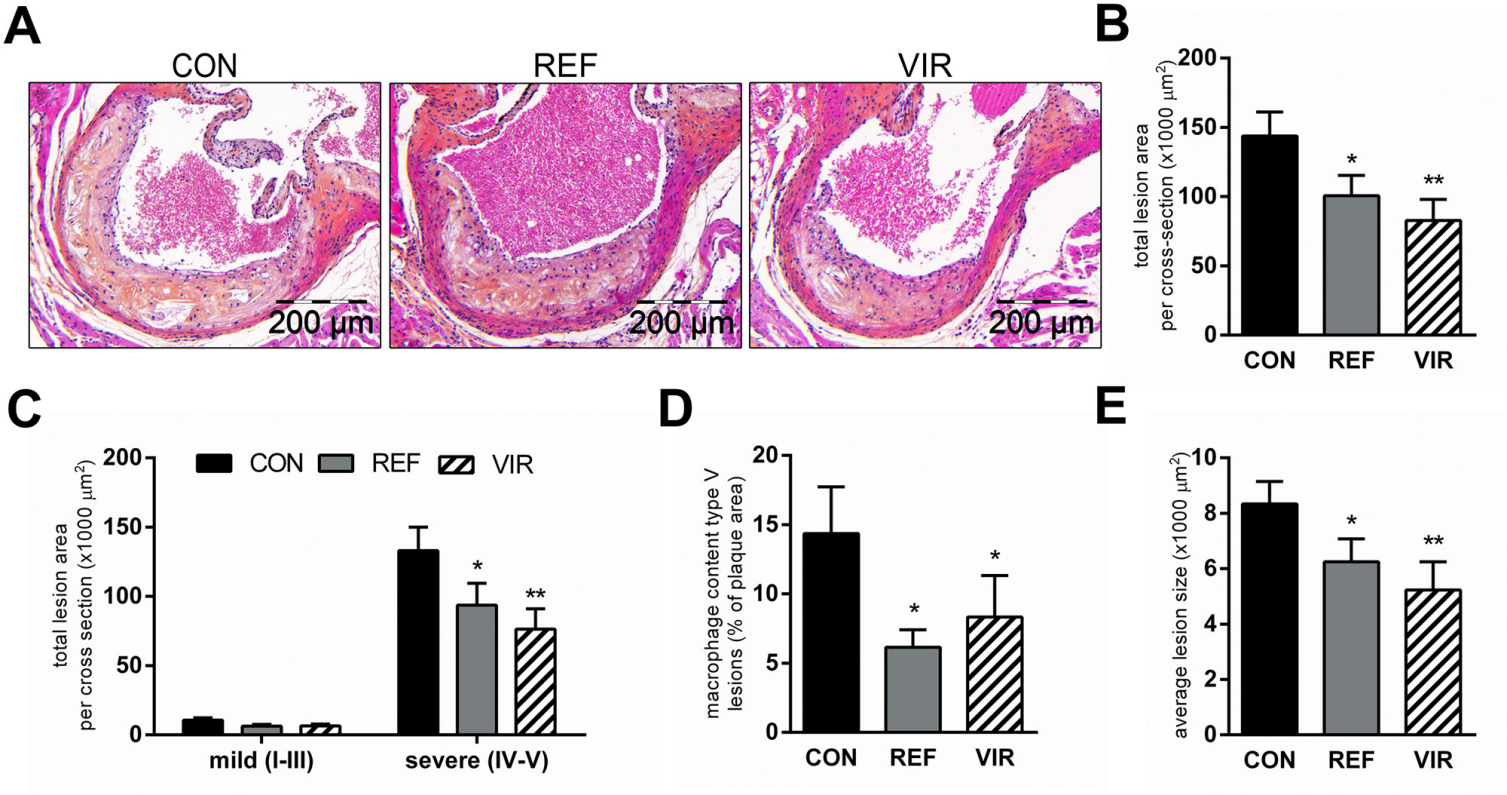
Atherosclerosis development is reduced with virgin pumpkin seed oil. Mice were fed a Western type control diet (CON) or CON diet containing 9% refined pumpkin seed oil (REF) or 9% virgin pumpkin seed oil (VIR) for 20 weeks. A: representative photomicrographs of HPS-stained cross sections of the aortic root show pronounced development of atherosclerosis in CON animals, which was less pronounced in REF and strongly reduced by VIR. B: Morphometric analysis of lesion area revealed a significant decrease in atherosclerotic lesion area by REF and VIR. C: Anti-atherogenic effects of pumpkin seed oils are specific on severe lesion types. D: Average lesion size was reduced in REF and VIR. E: Immunohistochemical staining for MAC-3 (CD107b) followed by quantification of positively stained area showed that both REF and VIR reduced the macrophage content of type V lesions. Data are mean±SEM. * p≤0.05, ** p≤0.01 compared with CON.

Atherosclerotic lesion area and number were quantified histologically in the valve area of the aortic root. CON diet induced pronounced atherosclerosis with a total lesion area of 143765±17286 μm^2^ per cross-section (Fig 4a and 4b). The total lesion area was reduced with REF (100594±14726 μm^2^, -30%, p≤0.05, Fig 4a and 4b) and an even stronger effect was observed in VIR (82766±15164 μm^2^, -42%, p≤0.01, Fig 4a and 4b). Refined morphological analysis of lesion severity revealed that the atherosclerotic lesion area in CON animals was mostly made up of large and advanced lesions (severe lesion types IV and V; Fig 4c). The observed decrease in total lesion area with REF and VIR was attributable to a significant reduction in the total area of these severe lesions specifically. Furthermore, immunohistochemical analysis of lesional macrophage content (MAC-3 positive area) showed that while there was no effect of REF or VIR on the macrophage content of mild type III lesions (not shown), the macrophage area in type V (severe) lesions was significantly reduced by both pumpkin seed oils (Fig 4d). In CON animals, 14.4±3.4% of the type 5 lesion area was MAC-3 positive and this was reduced to 6.16±1.26% in REF (p≤0.05 compared with CON) and 8.33±3.0% in VIR (p≤0.05 compared with CON). A similar, although non-significant, reduction was observed in type IV lesions (S3 Fig). The number of lesions (CON: 3.4±0.24; REF: 2.9±0.36; VIR: 2.6±0.21 lesions per cross-section; S3 Fig) and the percentage of lesion-free aortic segments (CON: 5.6±2.3; REF: 12.2±4.3; VIR: 9.5±3.3%; S3 Fig) were comparable among the groups. However the average size per lesion was significantly reduced by both oils (REF: -25%, p≤0.05; VIR: -37%, p≤0.01, Fig 4e), altogether indicating an effect on lesion growth rather than on the initiation of new lesions.

### Both pumpkin seed oils have beneficial effects on hepatic lipid metabolism while only virgin pumpkin seed oil reduces inflammation

To provide insight into the underlying processes modulated by VIR and REF, hepatic mRNA expression of genes involved in lipid metabolism and inflammation was analysed.

**Fig 5:**
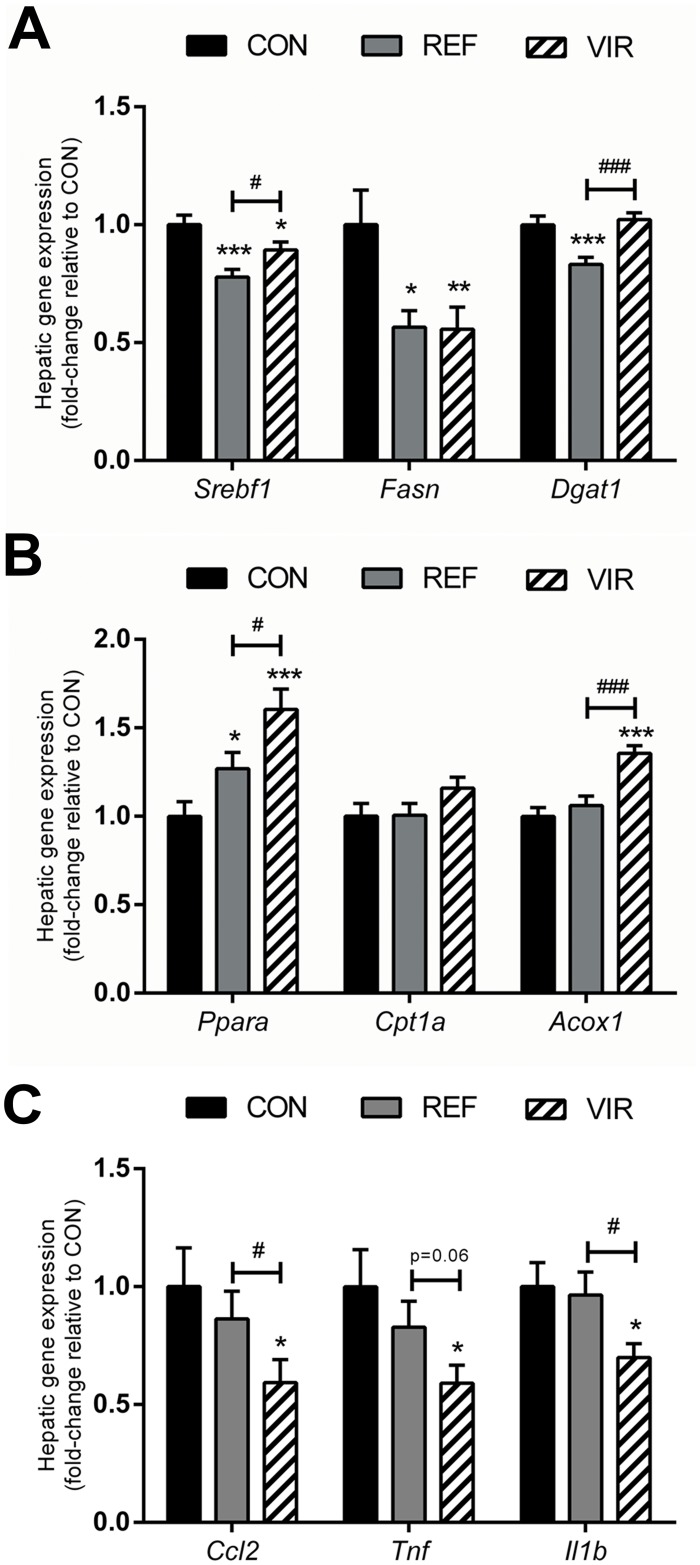
Refined and virgin pumpkin seed oils modulate lipid metabolism and inflammatory gene expression. Mice were fed a Western type control diet (CON) or CON diet containing 9% refined pumpkin seed oil (REF) or 9% virgin pumpkin seed oil (VIR) for 20 weeks. A: Hepatic lipogenic gene expression (*Srebf1*, *Fasn*, *Dgat1*) was reduced in both REF and VIR. B: Hepatic expression of genes involved in fatty acid catabolism (*Ppara*, *Cpt1a*, *Acox1*) was upregulated in VIR and to a lesser extent in REF. C. Only VIR reduced hepatic expression of inflammatory genes (*Ccl2*, *Tnf*, *IL1b*). All gene expression data are expressed as fold-change relative to CON. Data are mean±SEM. * p≤0.05, ** p≤0.01, *** p≤0.001 compared with CON, # p≤0.05, ### p≤0.001 for VIR compared with REF.

In line with the observed hypolipidaemic and antisteatotic effects of REF and VIR, expression of genes involved in lipogenesis was reduced by both pumpkin seed oils (Fig 5a). Expression of SREBP-1c (*Srebf1*), a master transcriptional regulator of *de novo* fatty acid and triglyceride synthesis [28] was reduced significantly in both REF (fold-change relative to CON: 0.78±0.03, p≤0.001) and VIR (0.89±0.03, p≤0.01). In line with this, the expression of the SREBP-1c target gene Fatty acid synthase (*Fasn*), the main biosynthetic enzyme in fatty acid synthesis [29], was also reduced in both REF (0.57±0.07, p≤0.05) and VIR (0.56±0.09, p≤0.01). Expression of Diacylglycerol acyltransferase-1 (*Dgat1*), which catalyses the final step in triglyceride synthesis [30], was significantly reduced in REF (0.83±0.03, p≤0.001), but unaffected in VIR. Together, these results provide indication that the *de novo* synthesis of lipids is reduced in pumpkin seed oil-fed animals.

Furthermore, mRNA expression analysis of genes involved in the catabolism of fatty acids (Fig 5b) revealed that pumpkin seed oil, particularly in its virgin form, may also stimulate the breakdown of lipids. Expression of Peroxisome proliferator activated receptor α (*Ppara*), the main regulator of β-oxidation [31] was increased in both REF (1.27±0.09, p≤0.05) and VIR (1.61±0.11, p≤0.001), with additional beneficial effects of VIR over REF (p≤0.05). Carnitine palmitoyl transferase I (*Cpt1a*), which catalyses the transport of fatty acids into the mitochondria [32] was not increased in REF (1.01±0.07) or VIR (1.16±0.06). Expression of Acyl-CoA oxidase (*Acox1)* which catalyses the first step of β-oxidation [33], was unaffected by REF (1.06±0.05), while it was significantly increased in VIR (1.36±0.04, p≤0.001). Altogether these results indicate a stimulating effect of VIR on β-oxidation while the effects of REF on this process appear to be less pronounced.

Investigation of hepatic inflammatory gene expression (Fig 5c) revealed an anti-inflammatory effect of VIR specifically, further strengthening the notion that phytochemicals in virgin pumpkin seed oil rather than the fatty acid composition of the oil *per se* are responsible for the observed anti-inflammatory effects. Expression of Monocyte chemoattractant protein-1 (*Ccl2*), which plays an important role in the recruitment of myeloid-derived monocytes [34] was not significantly affected by REF (0.86±0.12), while it was strongly reduced in VIR (0.59±0.10, p≤0.05). Similarly, expression of the pro-inflammatory cytokines Tumour necrosis factor alpha (*Tnfa*) and Interleukin 1 beta (*Il1b*) was significantly reduced by VIR (0.59±0.08, p≤0.05 for *Tnfa*; 0.70±0.06, p≤0.05 for *Il1b*) but not by REF (0.83±0.11 for *Tnfa*; 0.96±0.10 for *Il1b*).

## Discussion

In the study described herein, we demonstrate the potential long-term health effects of substitution of dietary fat (i.e. replacement of saturated by unsaturated fats), as well as putative additional effects of phytochemicals present in unrefined (virgin) oil. In a humanised model of disease, we show that both refined and virgin pumpkin seed oils markedly improve plasma lipids (cholesterol, triglycerides) and virgin pumpkin seed oil also reduced circulating markers of systemic and vascular inflammation. In the long run, both pumpkin seed oils attenuated the development of NAFLD and atherosclerosis, with a more pronounced effect of VIR in disease prevention.

Several epidemiological studies have shown that the development of NAFLD and CVD is associated with the type of dietary fat consumed [5–7]. To mimic diet-related long-term disease development in humans, we used the E3L model in which NAFLD and CVD are inducible by diet. These mice have a humanised lipoprotein profile, and cholesterol feeding results in a moderate elevation of plasma cholesterol (to about 18–20 mM) and combined development of NAFLD and atherosclerosis. Under the experimental conditions employed, lipid and inflammatory risk markers of future NAFLD and atherosclerosis are already induced after a few weeks, thus allowing the study of interventions on surrogate markers of disease, under conditions relevant for humans [17, 19, 23, 35].

Replacement of a part of the cocoa butter by pumpkin seed oil markedly diminished the induction of circulating risk factors (cholesterol, triglycerides, SAA, sVCAM-1), which is in line with the short-term effects of other pumpkin seed oil preparations tested in humans and animals [12, 14, 15]. As these studies employed different pumpkin seed oil preparations at different doses and treatment regimens (in capsules or by oral gavage, as an addition to the regular diet), they provide evidence for a general health benefit of pumpkin seed oil, independent of how it is prepared and administered (i.e. replacement of dietary fat, or on top of regular diet).

In the present study we exchanged a part of the main fat in the CON diet, which is cocoa butter (15% w/w of the diet), with pumpkin seed oil (9% w/w of the diet) which modifies the quality of fat consumed, without affecting the caloric density of the diet. More specifically, the main fatty acids present in cocoa butter are stearic acid (C18:0, 35.7%), palmitic acid (C16:0, 26.7%) and oleic acid (C18:1n-9, 32.8%), while linoleic acid (C18:2n-6, 2.7%) is only present in very small amounts. Replacing part of this cocoa butter with pumpkin seed oil, primarily increases the intake of linoleic acid and reduces the intake of oleic acid and the saturated fatty acids (SFA) stearic acid and palmitic acid. Linoleic acid is an essential n-6 poly-unsaturated fatty acid (PUFA) that is reported to have beneficial effects on plasma lipids (reviewed in [36]), in line with the results described herein. A possible rationale for the observed lipid-lowering effects may be found in activation of the transcription factor PPAR-α, which is known to be activated more strongly by PUFA than SFA [37]. Activation of this master regulator of lipid metabolism reportedly activates beta-oxidation in the liver and lowers plasma triglyceride levels as well as LDL cholesterol [38], consistent with observed reductions in plasma lipids in the present study. Gene expression analyses in the present study revealed an increased expression of PPAR-α in both pumpkin seed oil-fed groups, suggesting that transcriptional activity of this transcriptional regulator may be increased. Virgin pumpkin seed oil had additional effects on the expression and activation (demonstrated by increased expression of the PPAR-α target gene *Acox1*) of PPAR-α relative to the refined oil, indicating that phytochemicals present only in the virgin oil may have PPAR-α-activating properties. This is in line with findings by others, showing increased PPAR-α and PPAR-α target gene expression by tocopherols [39] and various polyphenol-rich mixtures (e.g. Apple polyphenols [40], Bilberry extract [41] and Walnut extract [42]). In contrast, there was no additional effect of the virgin oil on the reduction of lipogenic gene expression, thus indicating that these effects are attributable to the modification of the fatty acid composition of the diet, rather than effects of bioactive phytochemicals. More specifically, PUFAs are known to suppress SREBP-1c (the dominant transcriptional regulator of lipogenic genes) and rates of lipogenesis in rodents [43], in line with the effects of the PUFA-enriched pumpkin seed oil diets described herein. Remarkably, effects on lipogenic gene expression were more pronounced in the refined oil than in the virgin oil, suggesting that phytochemicals present in the virgin oil may attenuate these anti-lipogenic effects. Triglyceride and cholesterol-lowering effects comparable to those observed herein were also reported in long-term studies in E3L mice treated with long-chain PUFAs [44] or a PUFA-rich food supplement [45], as well as a pharmacological PPAR-α activator [23]. Overall, the reductions of plasma cholesterol achieved with the pumpkin seed oils are remarkably pronounced (-15% for REF, -24% for VIR). This effect is in the range typically achieved with low-doses of hypocholesterolemic drugs such as HMG-CoA reductase inhibitors (statins) in the E3L mouse as well as in patients [46, 47].

While both pumpkin seed oils had beneficial effects on dyslipidaemia, only VIR reduced markers of inflammation SAA and sVCAM-1, indicating that minor components that are present in VIR but not in REF may have anti-inflammatory properties. These anti-inflammatory effects may be conferred by specific phytochemicals, including polyphenolic compounds, of which virgin pumpkin seed oil is a rich source. The total phenolic content of the VIR preparation used in the present study was 8-fold higher than in REF. Polyphenols are widely recognised for their anti-inflammatory effects [48–50], and have frequently been reported to be protective against the development of NAFLD and cardiovascular disease, both in epidemiological and experimental studies [51, 52]. Under comparable experimental conditions and in the same mouse model, individual polyphenols were found to attenuate atherosclerotic lesion progression towards severe lesions [19, 35], which is consistent with the observed prevention of development of severe, vulnerable atherosclerotic lesions with pumpkin seed oil. Pumpkin seed oil contains a complex mixture of polyphenols and other bioactive phytochemicals and it is unlikely that observed beneficial effects are confined to a single phytochemical or one single mechanism. It is more likely that multiple bioactives affect multiple mechanisms (alone or in combination) that culminate in the net anti-inflammatory effects observed as has been demonstrated with other complex mixtures of bioactives [13, 45, 53–55].

Replacement of cocoa butter with pumpkin seed oil reduces the intake of palmitic acid by 50% (from 4% of total diet to 2% of total diet). Although palmitic acid is known to have pro-inflammatory effects on liver cells, the intake of this fatty acid was comparable in REF and VIR groups and can thus not explain the marked anti-inflammatory effects of VIR. However, it is likely that the increased intake in dietary PUFAs and the reduced intake of palmitic acid, as achieved with both oils, contributed to the reduction of liver inflammation as a marked (29%) decrease in inflammatory cell content was already observed with REF.

Overall, we show that a simple lifestyle modification, i.e. a switch in the type of fat consumed without reducing total fat or calorie intake, can make a significant contribution to reducing metabolic and cardiovascular disease risk. Partial replacement of the saturated fat-rich cocoa butter with refined pumpkin seed oil was sufficient to improve the risk factor dyslipidaemia, and affect development of NAFLD and atherosclerosis. Additional anti-inflammatory effects, conferred by minor components present only in the virgin oil, lead to profound reductions in disease endpoints. Importantly, the observed effects were achieved in a translational diet-induced disease model, with moderately increased plasma lipids and low-grade metabolic inflammation as is typical for high-risk populations in humans. Under these conditions, pumpkin seed oil represents a powerful means to improve dyslipidaemia, and, particularly when used in its virgin form, reduce chronic inflammation and prevent long-term disease development.

## Supporting Information

S1 Fig Refined and virgin pumpkin seed oils do not affect food intake or body weight in ApoE*3Leiden mice. Mice were fed a Western type diet (CON) containing 9% refined pumpkin seed oil (REF) or 9% virgin pumpkin seed oil (VIR) for 20 weeks. A: Average food intake was measured per cage in group-housed mice (3–4 mice per cage) and did not differ between groups. B: Body weight was not affected by either VIR or REF and increased gradually over time. Data are mean±SEM.
(TIF)

S2 Fig Refined and virgin pumpkin seed oils do not affect microvesicular steatosis in ApoE*3Leiden mice. Mice were fed a Western type diet (CON) containing 9% refined pumpkin seed oil (REF) or 9% virgin pumpkin seed oil (VIR) for 20 weeks. Microvesicular hepatosteatosis (% of total liver cross section affected) was not reduced by REF or VIR. Data are mean±SEM.
(TIF)

S3 Fig Refined and virgin pumpkin seed oils do not affect number of lesions or lesion-free segments ApoE*3Leiden mice. Mice were fed a Western type diet (CON) containing 9% refined pumpkin seed oil (REF) or 9% virgin pumpkin seed oil (VIR) for 20 weeks. A: Immunohistochemical staining for MAC-3 (CD107b) followed by quantification of positively stained area showed that the macrophage content of type IV lesions was not significantly reduced by REF or VIR. B: number of lesions per cross section were not reduced by REF or VIR. C: REF and VIR did not increase the percentage of lesion-free segments. Data are mean±SEM
(TIF)

S1 File ARRIVE Guidelines Checklist. (PDF)

S1 Text Detailed Material and Methods. (DOCX)
