# Supplementary figures and images for: Replacement of Dietary Saturated Fat by PUFA-Rich Pumpkin Seed Oil Attenuates Non-Alcoholic Fatty Liver Disease and Atherosclerosis Development, with Additional Health Effects of Virgin over Refined Oil
Source: PLoS One. 2015 Sep 25;10(9):e0139196. doi: 10.1371/journal.pone.0139196 (PMC4583328; doi:10.1371/journal.pone.0139196)

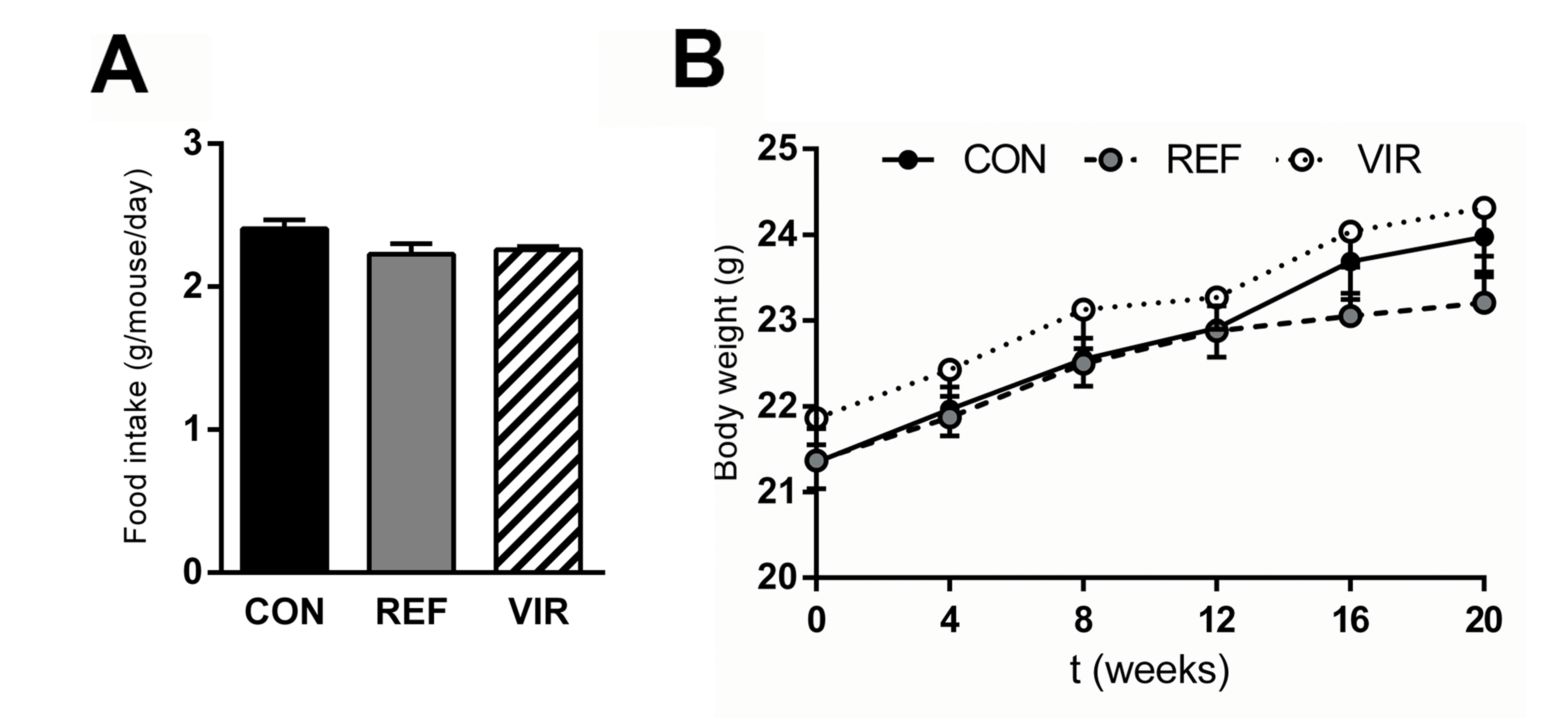

Supplement: S1 Fig — Mice were fed a Western type diet (CON) containing 9% refined pumpkin seed oil (REF) or 9% virgin pumpkin seed oil (VIR) for 20 weeks. A: Average food intake was measured per cage in group-housed mice (3–4 mice per cage) and did not differ between groups. B: Body weight was not affected by either VIR or REF and increased gradually over time. Data are mean±SEM. (TIF) [file pone.0139196.s001.tif]

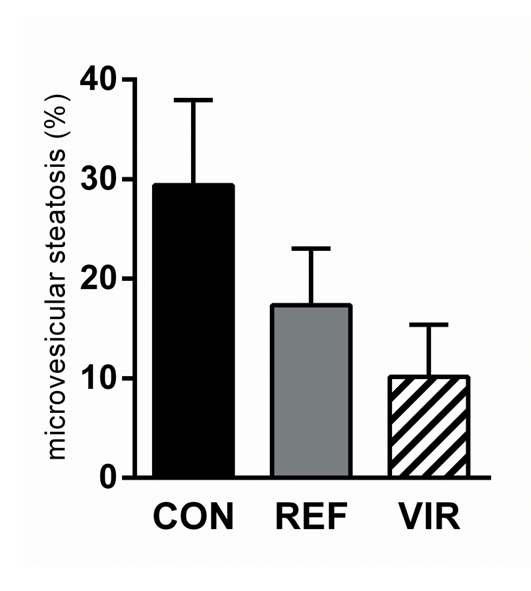

Supplement: S2 Fig — Mice were fed a Western type diet (CON) containing 9% refined pumpkin seed oil (REF) or 9% virgin pumpkin seed oil (VIR) for 20 weeks. Microvesicular hepatosteatosis (% of total liver cross section affected) was not reduced by REF or VIR. Data are mean±SEM. (TIF) [file pone.0139196.s002.tif]

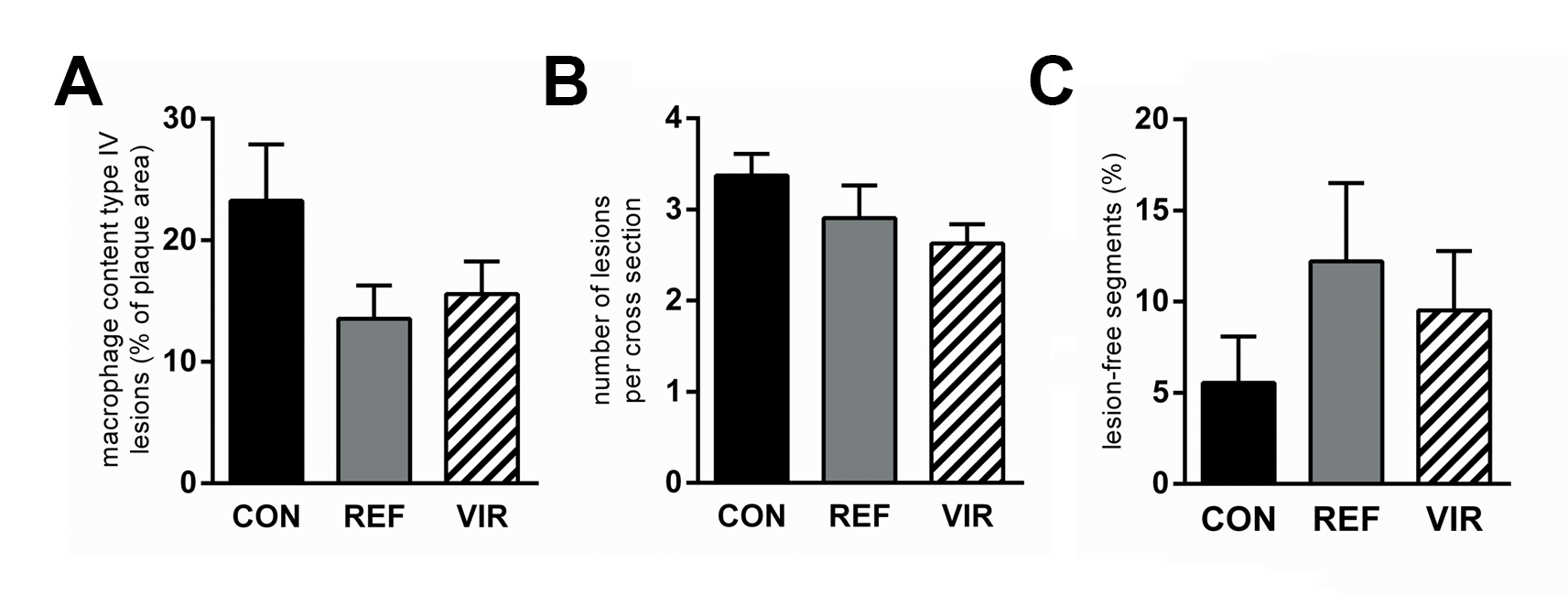

Supplement: S3 Fig — Mice were fed a Western type diet (CON) containing 9% refined pumpkin seed oil (REF) or 9% virgin pumpkin seed oil (VIR) for 20 weeks. A: Immunohistochemical staining for MAC-3 (CD107b) followed by quantification of positively stained area showed that the macrophage content of type IV lesions was not significantly reduced by REF or VIR. B: number of lesions per cross section were not reduced by REF or VIR. C: REF and VIR did not increase the percentage of lesion-free segments. Data are mean±SEM (TIF) [file pone.0139196.s003.tif]
