# Supplementary material for: Replacement of Dietary Saturated Fat by PUFA-Rich Pumpkin Seed Oil Attenuates Non-Alcoholic Fatty Liver Disease and Atherosclerosis Development, with Additional Health Effects of Virgin over Refined Oil
Source: PLoS One. 2015 Sep 25;10(9):e0139196. doi: 10.1371/journal.pone.0139196 (PMC4583328; doi:10.1371/journal.pone.0139196)
Supplement: S1 Text — (DOCX) [file pone.0139196.s005.docx]

**S1: Detailed materials and methods**

*Extraction of phenolic compounds from pumpkin seed oils*

A liquid-liquid extraction (LLE) was used to isolate the phenolic fraction of the cocoa butter and pumpkin seed oil samples, both refined and virgin. The extraction was carried out following the method described by Suarez et al. [1] with some modifications. Briefly, 20 mL of methanol:water (80:20, v/v) was added to 5 g of oil and homogenized for 2 min with a Ultraturrax (IKA Labortechnik). After that, two phases were separated by centrifugation at 637×g for 10min and the hydroalcoholic phase was transferred to a balloon. This step was repeated twice and the extracts were combined in the balloon. Then, the hydroalcoholic extracts were rotatory evaporated up to a syrupy consistency at 31 ◦C and were dissolved in 5 mL of acetonitrile. Afterwards, the extract was washed three times with 10 mL of n-hexane and the rejected n-hexane was treated with 5 mL of acetonitrile. The acetonitrile solution was finally rotatory evaporated to dryness and then re-dissolved in 1 mL of acetonitrile and maintained at −18 ◦C before the chromatographic analysis. 5 µL of the eluate was directly injected into the LC-QTOF-MS. Extractions were carried out in triplicate.

*LC-QTOF-MS analysis of phenolic extracts from pumpkin seed oils*

The analysis of the phenolic compounds and their metabolitesin the oil samples was carried out by means of a LC-QTOF-MS system consisted of a LC-Agilent 1290Series (Agilent Technologies, Palo Alto, U.S.A.) coupled to a 6540 ESI-QTOF (Agilent Technologies) operated in negative electrospray ionization mode (ESI-). Separation was carried out using a Zorbax SB-Aq column (3.5µm, 150mm x 2.1mm i.d.) equipped with a Pre-Column Zorbax SB-C18 (3.5µm, 15mm x 2.1mm i.d.) also from Agilent. Drying gas temperature was 350°C and the flow rate was held at 12 l/min. On the other hand pressure of the gas nebulizer was 45 psi and the capillary voltage was set at 4000 V. The fragmentor was set at 120V, the skimmer at 65V and the OCT 1RF Vpp was set at 750V.

During the analysis, the column was kept at 25°C and the flow rate was 0.4 mL/min. The solvent composition was solvent A: milli-Q water/acetic acid (99.8:0.2 v/v) and solvent B: acetonitrile. Solvent B was initially 5% and was gradually increased reaching 55% at 10 minutes and 95% at 12 min. Then it was maintained isocratically up to 15 min and after that it was reduced to 5% in 1 minute and was held at initial conditions during 8 minutes to re-equilibrate the column. The injection volume was set at 5 µL.

**References**

1. Suarez M, Macia A, Romero MP, Motilva MJ. (2008) Improved liquid chromatography tandem mass spectrometry method for the determination of phenolic compounds in virgin olive oil. J Chromatogr A 1214: 90-99.
